# Supplementary material for: Exploring the spatial variation and associated factors of childhood febrile illness among under-five children in Ethiopia: Geographically weighted regression analysis
Source: PLoS One. 2022 Dec 30;17(12):e0277565. doi: 10.1371/journal.pone.0277565 (PMC9803186; doi:10.1371/journal.pone.0277565)
Supplement: Attachment — Submitted filename: Response to Reviewers.docx [file pone.0277565.s003.docx]

**Point by point response for editors/reviewers comments**

**PLOSE ONE Journal**

**Manuscript title:** Exploring the Spatial Variation and Associated Factors of Childhood Febrile Illness among Under-Five Children in Ethiopia: A Geographically Weighted Regression Analysis

**Manuscript ID:**  PONE-D-22-11036

Dear editor/reviewer, we would like to thank you for the constructive, building, and improvable comments on this manuscript that would improve the content of the manuscript. We considered each comment and clarification question of editors and reviewers on the manuscript thoroughly. Our point-by-point responses for each comment and question are described in detail on the following pages. Further, the details of changes were shown by track changes in the supplementary document attached.

**Response to Editor**

1. Please ensure that your manuscript meets PLOS ONE's style requirements, including those for file naming. The PLOS ONE style templates can be found at <https://journals.plos.org/plosone/s/file?id=wjVg/PLOSOne_formatting_sample_main_body.pdf> and
   <https://journals.plos.org/plosone/s/file?id=ba62/PLOSOne_formatting_sample_title_authors_affiliations.pdf>

**Authors’ response:** Thank you Editor. We have modified the manuscript as per the PLOS ONE journal style requirements. **(See the revised manuscript)**

2. . We note that Figure 2, 4, 6, 7, 8 in your submission contain [map/satellite] images which may be copyrighted. All PLOS content is published under the Creative Commons Attribution License (CC BY 4.0), which means that the manuscript, images, and Supporting Information files will be freely available online, and any third party is permitted to access, download, copy, distribute, and use these materials in any way, even commercially, with proper attribution. For these reasons, we cannot publish previously copyrighted maps or satellite images created using proprietary data, such as Google software (Google Maps, Street View, and Earth). For more information, see our copyright guidelines: <http://journals.plos.org/plosone/s/licenses-and-copyright>.
    
   We require you to either (1) present written permission from the copyright holder to publish these figures specifically under the CC BY 4.0 license, or (2) remove the figures from your submission:
    
   1. You may seek permission from the original copyright holder of Figure 2, 4, 6, 7, 8 to publish the content specifically under the CC BY 4.0 license.

**Authors’ response:** Thank you dear Editor for your concern. The map is not copyrighted from somewhere rather we have done using GIS software based on the shapefile of Ethiopia received from Ethiopian Central Statistical Agency (CSA) by explaining the purpose of the study (accessed from Open-Africa shapefiles) and the GPS data (longitude and latitude) from measure DHS program by explaining the objective of the study through online requesting and allow us to access the shapefile and GPS data. Therefore, we kindly assure you that these figures are our results, produced using appropriate statistical approaches and procedure. We have published other studies which have these kinds of figures.

**Response to reviewers#1,2**

1. Is the manuscript technically sound, and do the data support the conclusions?

Both reviewers say “Yes”

2. Has the statistical analysis been performed appropriately and rigorously?

Both reviewers say “Yes”

3. Is the manuscript presented in an intelligible fashion and written in Standard English?

Both reviewers say “Yes”

**Response to reviewer#2**

**Author response:**

4. High prevalence found to be in Oromia region, due to the population in Oromia was high then high number of children’s involved in survey and mostly the areas were malaria endemic area. Therefore the prevalence was high.
5. The prevalence in figure-1 and hot spot area were – in figure-4, which is different because even though high prevalence in Oromia region but not hot spot area that is not found in clustered fashion. The hot spot area was found in Tigray region, south east of Amhara, border of north SNNPR and Oromia.
6. I was correct the word “whereas” as woreda
7. 10127 under five children were the total sample size for all clusters
8. Since the data was National survey in Ethiopia 2016 across all regions, no need of statistical calculation, simply the DHS data was extracted by weighting the data to be representative in all regions or across 643 clusters.
9. The tables, figures, words were revised to be satisfied PLOS ONE publication criteria.

Thank You!!!!
